# Acupuncture for Children with Cerebral Palsy: A Systematic Review Protocol

**DOI:** 10.2196/resprot.5944

**Published:** 2017-01-05

**Authors:** Taipin Guo, Bowen Zhu, Qian Zhang, Yejiao Yang, Qi He, Xinghe Zhang, Xiantao Tai

**Affiliations:** ^1^ School of Acupuncture-Tuina and Rehabilitation Yunnan University of Traditional Chinese Medicine Kunming China

**Keywords:** cerebral palsy, CP, children, acupuncture therapy, efficacy, safety

## Abstract

**Background:**

Cerebral palsy (CP), a childhood disease of high morbidity and serious harmfulness, has no effective therapies to completely relieve the associated pain. Acupuncture has been used widely in China to alleviate several CP symptoms, such as pain and motion disorders, despite the deficiency of high-quality evidence related to this practice.

**Objective:**

The aim of this systematic review protocol is to assess the efficacy and safety of acupuncture for the treatment of children with CP.

**Methods:**

The following electronic databases will be searched: Cochrane Library, Web of Science, EBASE, Springer, World Health Organization International Clinical Trials Registry Platform, China National Knowledge Infrastructure, Wan-fang database, Chinese Biomedical Literature Database, Chinese Scientific Journal Database, and other sources. All published randomized controlled trials from inception to December 2016 will be included. RevMan V.5.3 software will be implemented for the assessment of bias risk, data synthesis, subgroup analysis, and meta-analyses if inclusion conditions are met. Individuals recruited into the trials will include children with all types of CP, and these individuals will be involved as coresearchers to develop and evaluate the efficacy and safety of acupuncture for the treatment of children with CP. Due to language barriers, only English and Chinese articles will be retrieved.

**Results:**

The systematic review will synthesize the available knowledge surrounding acupuncture for children with CP. The findings will be synthesized to determine the efficacy and safety of acupuncture for children with CP.

**Conclusions:**

The review has not been completed. This protocol presents a proper method to implement the systematic review, and ensures transparency for the completed review. Findings from the systematic review will be disseminated in a peer-reviewed journal and results will be presented at relevant conferences. The data of individual patients will not be included, so ethical approval is not required.

**Trial Registration:**

PROSPERO registration number: CRD42016038275, http://www.crd.york.ac.uk/PROSPERO/display_record.asp?ID=CRD42016038275 (Archived by WebCite at http://www.webcitation/6nGxoJrqm

## Introduction

Cerebral palsy (CP), the most common chronic disability of childhood, encompasses a heterogeneous group of movement and posture disorders that originate in the developing fetal or infant brain [1,2]. Epidemiological studies have found that the global prevalence of CP is 1.5-4 per 1000 live births, and the prevalence is higher in poor areas [3,4]. For example, with a prevalence of 3.6 per 1000 newborns in the United States, more than one hundred thousand children are affected in this country alone [5]. Considering the parents and caretakers of these children, the number of people who are negatively impacted by CP is far more considerable than that the patients themselves.

As an incurable illness, CP not only brings significant economic burdens to families (an average lifetime cost of one million dollars per person in the United States), but the disease if also associated with additional psychological problems [6,7]. Compared to healthy individuals, CP is associated with a higher frequency of autism spectrum disorders, sclerotic deformity, and the incidence of fractures [8,9].

Considering the complex manifestations of CP, treatments are targeted at improving function and reducing disability. Current treatments include pharmacological interventions, surgical interventions, physical and behavioral therapy, mechanical aids, and management of associated medical conditions. Although these commonly used clinical treatments of CP are multitudinous, none have accomplished a satisfying curative effect [3].

Acupuncture has increasingly been integrated into pediatric health care [10], and although numerous studies have been published, the safety and efficacy of this CP treatment is still ambiguous. Previous systematic reviews regarding acupuncture showed pain relief descriptively, and none included quantitative analyses for specific outcomes, leading to controversial results [11,12]. As more studies examining acupuncture for CP are performed, systematic reviews updated in 2008 or 2009 have become obsolete [13].

In traditional Chinese medicine (TCM), acupuncture therapy has been used for thousands of years, and has incorporated clinical treatment experiences to fine tune the procedures [14]. The theory of TCM suggests that health is achieved by maintaining an uninterrupted flow of Qi. Qi flows through a network of 14 channels, called *meridians*, which run along the surface of the human body. The acupuncture needles insert into the specific pathways or meridians at specific angles to correct the imbalance of energy in the body and restore natural internal homeostasis [15].

The theory of TCM deems that acupuncture is thought to correct the imbalance of energy in the body and restore natural internal homeostasis by stimulating various meridian points. At specific points, the needle inserts into the skin for dredging stasis in the meridian to adjust Yin and Yang. Studies have demonstrated that scalp acupuncture therapy may have the potential to treat epilepsy by increasing the blood flow speed of microchannel architecture, and upregulate anticardiolipin levels [16]. Decreased expression levels of cystathionine beta-synthase, and increased expression levels heme oxygenase-1 and hypoxia-inducible factor-1α, have been observed in perinatal rat cortex cells after electrical acupuncture treatment, implicating a novel protective mechanism for CP [17]. Furthermore, some studies indicate a high rate of symptom improvement in patients with CP after acupuncture treatment [18-21]. However, the safety and effect of acupuncture is not clear. The goal of this systematic review is to estimate the effectiveness and safety of acupuncture on CP, and to formulate treatment recommendations.

## Methods

### The Systematic Review

All Chinese and English randomized controlled trials (RCTs) published in electronic databases from inception to October 2016 will be included in this review. If inclusion criteria are met, we will use RevMan V.5.3 software to assess the risk of bias, examine data synthesis, undertake subgroup analysis, and conduct meta-analyses.

### Electronic Search Strategy

Relevant databases include: Cochrane Library, Web of Science, EBASE, Springer, World Health Organization International Clinical Trials Registry Platform, China National Knowledge Infrastructure, Wan-fang database, Chinese Biomedical Literature Database, and Chinese Scientific Journal Database. The search strategy will be formulated in accordance with the guidance provided by the Cochrane Handbook [22]. The Medline search strategy is listed in Table 1, which includes all search terms, and other searches will be conducted based on these results.

### Screening and Selection Criteria

#### Overview

**Figure 1:**
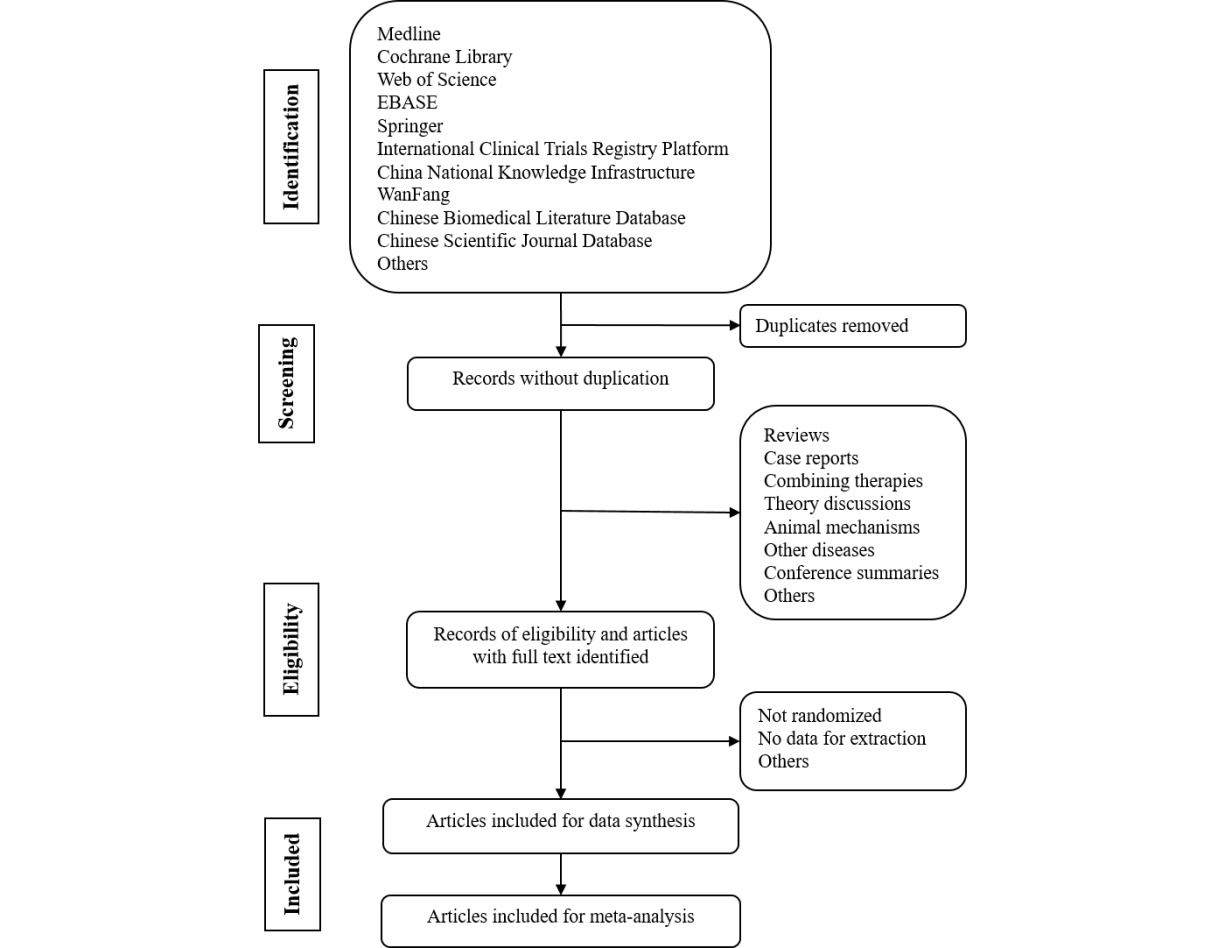
Medline search strategy.

Two authors (ZBW and ZQ) will individually screen the title and abstract of each publication. Articles that clearly do not meet the inclusion criteria, and those that are not relevant to the study, will be excluded. Disagreements or discrepancies related to the inclusion criteria will be resolved through discussion. The screening process is summarized in Figure 1.

**Table 1:** Medline search strategy.

Number	Search terms
1	Randomized controlled trial
2	Controlled clinical trial
3	Randomly
4	Randomised
5	Randomized
6	Trial
7	or/1-6 (“or” is a representative of connecting two coordinating relation words)
8	Cerebral palsy
9	CP
10	Brain paralysis
11	Feilian
12	Encephala paralysis
13	or/8-12
14	Acupuncture
15	Acupoint
16	Meridian
17	Electro-acupuncture
18	Electroacupuncture
19	Transcutaneous electrical nerve stimulation
20	Acupoint catgut embedding
21	Acupressure
22	Cupping jar
23	Moxibustion
24	Auricular points
25	Abdominal acupuncture points
26	Scalp acupuncture points
27	Laser
28	Magnets
29	Bleeding
30	Acupoint injection
31	Fire needle
32	Needle-knife
33	Superficial needling
34	or/14-33
35	7 and 13 and 34

#### Intervention

Several types of acupuncture will be included in the review: electro-acupuncture, body acupuncture, scalp acupuncture, ear acupuncture, fire needling, laser acupuncture, transcutaneous electrical nerve stimulation, and point injection. Multiple control interventions will also be included: no treatment, placebo/sham acupuncture, and other interventions (eg, surgery, drugs, and physical interventions). We will also include trials that evaluate acupuncture in addition to other therapies, if these combinations are compared to the other therapy alone.

#### Comparisons

If possible, we will execute the following analyses: (1) comparisons between acupuncture and sham, placebo, or no treatment; (2) comparisons between acupuncture and pharmacological interventions; and (3) comparison between acupuncture and physical interventions.

Review Manager 5.3 will be employed to test for interactions. When considering different types of CP, we will use a graphical display to assess each one separately for the curative effect and safety of acupuncture. Subgroup analyses will be performed between the different types of acupuncture.

#### Primary and Secondary Outcomes

Primary outcomes will include (1) motor function improvement; (2) intellectual development; (3) improvement of self-care ability and daily living; and (4) side effects of acupuncture. Secondary outcomes will include (1) symptom improvement; (2) quality of life, self-esteem, and self-concept development; and (3) satisfaction with the treatment.

### Study Design

#### Inclusion Criteria

We will examine RCTs that involve at least one test treatment that aimed to improve or eliminate CP symptoms, and one control treatment (or no treatment) with concurrent enrolment. Study participants must conform to diagnosis standards established by bodies such as the American Academy of Neurology or the American Association of Cerebral Palsy. All children <18 years of age will be included in the study, with no limits on the ethnicity, nationality, type of CP, or gender of the subjects.

#### Exclusion Criteria

Trials comparing different types of acupuncture will be excluded. Participants that did not have a specific diagnosis of pediatric CP will be excluded. Patients with motor development diseases, diseases of bones and muscles, common genetic diseases, spinal cord diseases, and peripheral neuropathy will be excluded.

#### Date Extraction

Using a standalone electronic form, two review authors (YYJ and HQ) will extract data independently and examine the publication date of each report, to minimize errors and reduce potential risk of bias. The information of participants, interventions, controls, and outcomes will be extracted. Disagreements will be resolved through discussion.

#### Quality Assessment

Depending on the sample size of each study, and the influence of pooled effect size and strength of evidence, discussions will be undertaken to determine if the study will be included. Two authors (ZBW and GTP) will assess the risk of bias based on the domains and criteria of the Cochrane Collaboration’s tool [22]. Six domains of bias will be examined: (1) selection bias, (2) performance bias, (3) attrition bias, (4) detection bias, (5) reporting bias, and (6) other biases.

Any problems or disagreements will be discussed with a third author (GTP). For dichotomous data, we will present a risk ratio (RR) with 95% CIs. For continuous outcomes, we will present a standard mean difference with 95% CI. Other binary data will be changed into the RR form. If missing data is evident in any of the studies, we will attempt to contact the authors by phone or email. If the missing data are not obtained, the available data will be analyzed with the assumption that it is missing at random. If necessary, missing data will be imputed using replacement values.

A standard χ2 statistic and I^2^statistic will be used to measure heterogeneity among trials. If there is no statistical heterogeneity observed between subgroups (I^2^<50%), heterogeneity will be accepted. If substantial heterogeneity exists (I^2^>50%), indicating a level of inconsistency, we will examine possible causes of heterogeneity. If a sufficient number of studies are available (a minimum of 10 are required), we will use funnel plots to detect the reporting biases.

### Data Synthesis

If two or more eligible RCTs are identified, we will perform meta-analyses with Review Manager 5.3. When I^2^<50%, the fixed-effect model will be chosen, and the random-effect model will be selected I^2^>50%. We will explore possible causes and analyze the data with a random-effect model. If clinical and methodological heterogeneity is presented, we will perform subgroup analyses; if not, we will not pool the data and a systematic narrative synthesis will be performed to summarize and explain the characteristics and findings of the included studies.

## Results

This systematic review aims to evaluate the effectiveness and safety of acupuncture for CP in children, and assist with future research planning. By synthesizing included studies, the systematic review results will provide insights regarding which components of interventions are most effective and safe. The completion date for the review is projected to be early-to-mid 2017.

## Discussion

CP is a disease with complex pathogenesis and brings huge burdens to the patients and their families [23]. TCM has long-standing and abundant experiences in treatments for children with CP. As an important therapy of TCM, acupuncture might also play an important role in the treatment of all CP cases. Systematic reviews show that acupuncture is a safe intervention for pediatric patients, and no severe side effects have been reported [12,24].

A systematic review of acupuncture usage in the treatment of CP should provide a reliable basis of evidence, and expand the understanding of acupuncture in CP. Although acupuncture is extensively used in the treatment of children in clinics, adverse events of acupuncture are often ignored or not reported in reviews [13]. In addition to evaluating the efficacy of acupuncture, this systematic review will also examine the safety of acupuncture in pediatric health care by evaluating the safety of acupuncture for children with CP.

Due to language barriers, we will only retrieve English and Chinese articles, which may affect the strength of results. Two researchers will independently examine the articles to eliminate personal biases related to study selection, data extraction, and quality assessment. To evaluate the safety and efficacy of pediatric CP, the protocol will be carried out to explore any sources of heterogeneity in different acupuncture therapies.

This study will evaluate the efficacy and safety of acupuncture for the treatment of children with CP. This protocol presents a proper method to implement the systematic review, and ensures the transparency of the completed review.

## Abbreviations

CP: cerebral palsy
RCT: randomized controlled trial
RR: risk ratio
TCM: traditional Chinese medicine
